# How Copepods Can Eat Toxins Without Getting Sick: Gut Bacteria Help Zooplankton to Feed in Cyanobacteria Blooms

**DOI:** 10.3389/fmicb.2020.589816

**Published:** 2021-01-12

**Authors:** Elena Gorokhova, Rehab El-Shehawy, Maiju Lehtiniemi, Andrius Garbaras

**Affiliations:** ^1^Department of Environmental Science and Analytical Chemistry, Stockholm University, Stockholm, Sweden; ^2^Marine Research Centre, Finnish Environment Institute (SYKE), Helsinki, Finland; ^3^Mass Spectrometry Laboratory, Center for Physical Science and Technology, Vilnius, Lithuania

**Keywords:** nodularin, microcystin, copepods, *mlrA* gene, biodegradation, hepatotoxins, grazing, growth

## Abstract

Toxin-producing cyanobacteria can be harmful to aquatic biota, although some grazers utilize them with often beneficial effects on their growth and reproduction. It is commonly assumed that gut microbiota facilitates host adaptation to the diet; however, the evidence for adaptation mechanisms is scarce. Here, we investigated the abundance of *mlrA* genes in the gut of the Baltic copepods *Acartia bifilosa* and *Eurytemora affinis* during cyanobacteria bloom season (August) and outside it (February). The *mlrA* genes are unique to microcystin and nodularin degraders, thus indicating the capacity to break down these toxins by the microbiota. The *mlrA* genes were expressed in the copepod gut year-round, being >10-fold higher in the summer than in the winter populations. Moreover, they were significantly more abundant in *Eurytemora* than *Acartia*. To understand the ecological implications of this variability, we conducted feeding experiments using summer- and winter-collected copepods to examine if/how the *mlrA* abundance in the microbiota affect: (1) uptake of toxic *Nodularia spumigena*, (2) uptake of a non-toxic algal food offered in mixtures with *N. spumigena*, and (3) concomitant growth potential in the copepods. The findings provide empirical evidence that the occurrence of *mlrA* genes in the copepod microbiome facilitates nutrient uptake and growth when feeding on phytoplankton mixtures containing nodularin-producing cyanobacteria; thus, providing an adaptation mechanism to the cyanobacteria blooms.

## Introduction

The expansion of harmful cyanobacteria blooms and associated risks for environmental health is a matter of concern (O’Neil et al., 2012). In the Baltic Sea, cyanobacteria blooms are a natural phenomenon; however, in line with the global trends, the magnitude and duration of these blooms increase (Kahru and Elmgren, 2014). The filamentous diazotrophic cyanobacteria *Nodularia spumigena* Mertens, *Aphanizomenon* sp., and *Dolichospermum* (formerly *Anabaena*) spp. are the major contributors of the Baltic cyanobacterial blooms (Wasmund, 1997). As most cyanobacteria, these species produce numerous bioactive compounds and toxins (Sivonen, 2009) and are considered potentially harmful (Karjalainen et al., 2007). *Aphanizomenon* sp. and *Dolichospermum* spp. are known to produce hepatotoxic microcystins in freshwaters (Sivonen and Börner, 2008); moreover, Baltic *Dolichospermum* can also produce microcystins (Halinen et al., 2007). However, of main concern is *N. spumigena*, a well-known producer of hepatotoxin nodularin, which makes up to∼90% of cyano-hepatotoxins in the Baltic Sea (Kankaanpää et al., 2009).

In the environment, microbial degradation is the main route for cyanotoxin breakdown by heterotrophic bacteria (Jones et al., 1994; Cousins et al., 1996; Okano et al., 2009). Both microcystins and nodularins released during a bloom decay are usually degraded within several days (Christoffersen et al., 2002; Tuomainen et al., 2006; Dziga et al., 2019). The main focus has been on microcystin degraders driven by the *Microcystis* blooms in freshwaters and the widespread use of freshwater lakes and reservoirs for drinking water supply (Ho et al., 2012). However, some microcystin-degraders were also found to degrade nodularin (Harada et al., 2004). The most studied microcystin- and nodularin-degraders belong to genera *Sphingomonas* and *Sphingopyxis* and some other genera of the family Sphingomonadaceae (Jones et al., 1994; Park et al., 2001; Imanishi et al., 2005; Valeria et al., 2006; Yan et al., 2012). The list of isolated bacterial strains capable of degrading microcystin is growing and mainly include members belonging to phylum Proteobacteria but also Firmicutes and Actinobacteria (Dziga et al., 2013; Li et al., 2017), and it is apparent that the capacity to degrade microcystin and nodularin is spread across bacterial phyla (Toruńska-Sitarz et al., 2018).

Under aerobic conditions, the microcystin and nodularin degradation is driven by more than one enzymatic pathway (Dziga et al., 2013, 2019; Toruńska-Sitarz et al., 2018); however, only one specific pathway has been characterized so far. The cluster designated mlrABCD encodes a microcystin degradation pathway, in which a microcystinase (MlrA) is the first enzyme to hydrolyze cyclic microcystin into a linear intermediate. The resulting linear molecule has significantly reduced toxicity (Bourne et al., 1996). The highly conserved *mlrA* gene is unique to microcystin degraders but not to the family Sphingomonadales, where it was first described (Dziga et al., 2013). To quantify microcystin-degrading bacteria that possess this gene, a qPCR assay targeting the *mlrA* gene was developed (Hoefel et al., 2009) and used to estimate the abundance of *mlrA* as a proxy for the *in situ* capacity for biodegradation of microcystin via the mlrA-D pathway (Zhu et al., 2014; Lezcano et al., 2017).

Cyanobacterial blooms can exert various impacts on aquatic biota. While laboratory studies often report adverse effects on zooplankton grazers resulting from cyanobacteria nutritional inadequacy and toxicity, high zooplankton biomass is frequently observed during cyanobacterial blooms in freshwater lakes and the Baltic Sea (Hogfors et al., 2014; Karlson et al., 2015). The high population abundance of grazers coinciding with blooms reflects that cyanobacteria are consumed by many grazers, suspension-feeders, and deposit-feeders, often with beneficial effects on their growth and reproduction (Karlson et al., 2015; Motwani et al., 2017). Keeping in mind that Baltic fauna and cyanobacteria have co-evolved over the past 7,000 years, it is likely that grazers exposed to these blooms have adaptive strategies to optimize their energy and nutrient intake despite the diverse (and evolving) arsenal of cyanobacterial toxins and various bioactive compounds. These strategies may include biochemical adaptations (e.g., enzymes that counteract toxins, such as mixed-function oxidases and Cytochrome P450 oxidases), behavioral adaptations (e.g., grazing avoidance and escape reactions, if toxin concentrations increase beyond a critical level), and microbial symbionts (e.g., gut microbiota capable of breaking down or inactivating toxins). None of these adaptations are mutually exclusive, and they may act together to yield a phenotype capable of utilizing cyanobacteria as a food source with minimal fitness penalties.

Diet plays a central role in shaping the microbiota in eukaryotes, including arthropods (Karasov et al., 2011). Crustaceans harbor diverse microbial communities consisting of bacteria, viruses, and fungi inhabiting internal and external body surfaces. The host nutrition is crucially dependent on the gut microorganisms that provide certain nutrients, vitamins, and maintain the gut digestive capacity (Harris, 1993). The adaptation of the gut microbiota to the diet is often species-specific and may respond to seasonal variability in consumer physiology as well as resource availability and quality (Tang et al., 2009; Bolaños et al., 2016). Microbes can bring innovations to their hosts, allowing the exploitation of new or inhospitable environments (often referred to as ecological opportunities), including challenging food resources, such as hepatotoxic cyanobacteria. In terrestrial arthropods, metagenomic profiles showed various toxic compound degradation pathways suggesting that gut microbiota is adaptable to dietary toxins and bioactive compounds (Bolaños et al., 2016).

We hypothesized that planktonic copepods commonly co-occurring with the cyanobacterial blooms in the Baltic Sea can cope with their toxicity because they harbor microcystin/nodularin degrading bacteria in their microbiome. Consequently, the copepod capacity to utilize hepatotoxic cyanobacteria would be positively related to the abundance of *mlrA*-carrying bacteria in the host microbiome. As cyanobacteria blooms occur only in summer, we expected to find higher *mlrA* levels in the summer copepod populations exposed to the bloom than those in winter and a higher capacity of the host to utilize a cyanobacteria-containing diet in the summer compared to the winter populations. To test these relationships, we conducted (1) a survey to investigate whether *mlrA* genes are present in the copepod microbiota, and (2) feeding experiments using summer- and winter-collected copepods exposed to cyanobacteria.

## Materials and Methods

### Test Species

The copepods *Acartia bifilosa* and *Eurytemora affinis* are the dominant mesozooplankton species in the Northern Baltic Proper (Johansson et al., 2004) and a common prey for the main zooplanktivores (Hansson et al., 1990). When selecting test specimens in the field survey and feeding experiments, we focused on the older copepodites, CIV-V, females, standardizing sample mass, avoiding the appearance of eggs/nauplii in the feeding trials, and minimizing variability due to ontogenetic development.

### Field Survey

Within the Swedish National Marine Monitoring Programme (SNMMP) and various research projects, the copepod samples were collected during the growth season (June to September) at several stations in the Northern Baltic proper (Table 1; for details, see Supplementary Table S1). This collection was complemented with winter samples collected in February 2016 onboard R/V *Ararnda* (SYKE, Finland). Copepods were collected by vertical hauls with WP2 net (90 or 100 μm), preserved in RNA*later* (Gorokhova, 2005), and stored at −20°C until analysis.

**TABLE 1:** Summary of *Acartia bifilosa* and *Eurytemora affinis* samples used for the analysis of *mlrA* gene abundance (A) and locations where the animals were collected for the feeding experiments (B).

**Station**	**Location and bottom depth**	**Geographic coordinates**	**Month, year**	**Number of samples**	
				***A. bifilosa***	***E. affinis***
**(A)**					
B1	Askö station, WGB; 40 m	N58°48′18, E17°37′52	Jun–Sep, 2010–2011	15	14
H4	Himmerfjärden Bay, WGB; 30 m	N58°59′02,E 17°43′50	Jul, 2009	3	4
BY31	Landsort Deep, WGB; 454 m	N60°11.34′ E19°08.55′	Jun–Sep, 2011	21	23
F69/winter	NBP; 191 m	N59.4700 E019.5580	Feb, 2016	4	3
BY32/winter	WGB; 169 m	N57.5999 E017.5981	Feb, 2016	3	4
AALTOPI/winter	NBP; 98 m	N59.1502 E020.5982	Feb, 2016	3	3
**(B)**					
B1/summer	Askö station, WGB; 40 m	N58°48′18, E17°37′52	Aug, 2014	14	13
F69/winter	NBP; 191 m	N59.4700 E019.5580	Feb, 2016	4	3
BY32/winter	WGB; 169 m	N57.5999 E017.5981	Feb, 2016	3	4
AALTOPI/winter	NBP; 98 m	N59.1502 E020.5982	Feb, 2016	3	3

*Station names are given according to the SHARK and SYKE databases; Baltic Sea basins: NBP, Northern Baltic Proper; WGB, Western Gotland Basin. The number of samples corresponds to the total number of samples per species per station analyzed by the qPCR assay; each sample for mlrA gene analysis contained 9–10 individuals. See Supplementary Table S1 for the data on cyanobacteria and mlrA gene abundance recorded on each sampling occasion.*

The summer data on filamentous cyanobacteria carbon biomass (μg C L^–1^) corresponding to the time of the copepod sampling (i.e., collected within a week) were retrieved from Swedish Meteorological and Hydrological Institute; SHARK database)^1^ for stations B1 and BY31, and from Himmerfjärden Eutrophication Study^2^ for station H4. The total carbon biomass values for *Nodularia spumigena* and *Dolichospermum* sp. were calculated; in the study area, these are the only taxa reported to produce nodularin and microcystin, respectively. During the winter sampling, none of these cyanobacteria were observed when examining plankton communities onboard, and none were present in the corresponding monitoring records; therefore, their carbon biomass in the winter period of the study was assumed to be zero.

### Feeding Experiments

Based on the positive relationship between the *mlrA* occurrence in the copepod samples and cyanobacteria biomass in the ambient communities derived from the survey data, we hypothesized that *mlrA* gene abundance is related to the copepod capacity to feed on cyanobacteria when provided in mixtures with edible phytoplankton and to utilize it for growth. To test our hypothesis, we studied whether the *mlrA* abundance in the copepod microbiome is related to: (1) uptake of *Nodularia spumigena*; (2) uptake of high-quality algal food (*Rhodomonas salina*) offered in mixtures with *N. spumigena*; and (3) growth potential in the copepods. We used dual-isotope labeling, ^13^C and ^15^N, for *N. spumigena* and *R. salina*, respectively, to trace the uptake of cyanobacteria and algae by the copepods.

The feeding experiments were conducted on two occasions: August 2014 (summer populations) and February 2016 (winter populations). For the summer experiment, the copepods were collected in the vicinity of Askö Field Station and transported in insulated containers to the laboratory, where the experiment was conducted. The winter experiment was conducted onboard R/V *Aranda* during a research cruise in the Baltic proper (Table 1). For acclimation and purging gut from the ambient food, the experimental animals were transferred to 0.2-μm filtered seawater and provided with unlabeled axenic *R. salina* at the concentration of 8 × 10^6^ cell mL^–1^ twice a day. After 12-h acclimation, we sampled specimens of both species to measure: (1) abundance of the *mlrA* genes (9–10 ind. species^–1^); and (2) stable isotope signatures and mean body weight (30 ind. species^–1^). All samples were preserved in RNA*later* (Gorokhova, 2005) at the start of the experiment.

As experimental food, we used *Nodularia spumigena* (Cyanophyceae; strain AV1) and *Rhodomonas salina* (Cryptophyceae; CCMP 1319) grown in Z8 and f/2 media, respectively, at 8 PSU (Practical Salinity Units). The AV1 strain of *N. spumigena* was isolated from the open Gulf of Finland (Sivonen et al., 1989). It is a potent nodularin producer with relatively stable intracellular and extracellular nodularin concentrations during a 3-weeks growth period (Jonasson et al., 2008). The intracellular nodularin concentrations in the ^13^C-labeled and unlabeled cultures were analyzed by ELISA, using a microcystin plate kit (EnviroLogix, Portland, ME, United States) with nodularin standards (0.08–1.8 ng mL^–1^). The sample preparation and analysis workflow are described elsewhere (Mathys and Surholt, 2004). In brief, the cultures were collected on GF/F filters, deep-frozen, lyophilized, and extracted in 75% methanol by sonicating for 30 min. The extraction procedure was repeated twice, and the supernatants were combined and dried under N_2_ flow. The samples were then re-suspended in 75% methanol under sonication; sequential dilutions were prepared, dried under N_2_ flow, and re-suspended in 0.9% NaCl before loading on the ELISA plate. The spectrophotometric analysis followed the ELISA kit manufacturer guidelines; the absorbance was measured at 450 nm with a microplate spectrofluorometer (FLUOstar OPTIMA, BMG LabTechnologies). The sample nodularin concentrations were calculated using a semi-logarithmic function by plotting calibrators and negative control results. Following this procedure, the average nodularin concentration in the stock culture was 60 μg mg Nodularia dry weight^–1^, with no significant differences between the ^13^C-labeled and unlabeled cultures and between the summer and winter experiments (Supplementary Figure S1).

To label algal cultures with ^13^C and ^15^N, we replaced NaH^12^CO_3_ with NaH^13^CO_3_ and Na^14^NO_3_ with Na^15^NO^3^ when preparing the growth medium. In this way, 3% of the NaHCO_3_ and NaNO_3_ were enriched with ^13^C and ^15^N, respectively (98 atom % pure, Aldrich, St. Louis, MO, United States). The algae were grown at 17°C, and the illumination of ∼40 μE ⋅ m^–2^⋅s^–1^ with constant shaking. The isotope-enriched solutions (Andriukonis and Gorokhova, 2017) were added 6 and 1.5 days before harvesting, for nitrate and bicarbonate, respectively, to ensure sufficient uptake. The labeling procedure yielded stocks of *N. spumigena* and *R. salina* with the isotopic values of δ^13^C = 6754 ‰ and δ^15^N = 1095 ‰, respectively, for the summer experiment and δ^13^C = 5160 ‰ and δ^15^N = 1186 ‰, respectively, for the winter experiment. To prepare the experimental diets, the labeled and unlabeled cyanobacteria/algae were mixed to produce 2 at% of ^13^C and 1 at% of ^15^N. After labeling, the algal cells were collected and washed on a pre-combusted (450°C for 5 h) 25-mm diameter glass fiber filter (Whatman type GF/C), washed with deionized water to remove unassimilated isotopes, and re-suspended in distilled water. The cultures used in the food mixtures were pre-treated with gamma-radiation (Gorokhova et al., 2012) to prevent cell division, minimize algal growth during the experiment, and increase the accuracy of food consumption estimates (Gorokhova et al., 2013). The algal concentrations (cells mL^–1^) were determined for each experimental food batch at the start using a laser particle counter Spectrex PC-2000 (Spectrex Corp., California). The final stocks were kept at 8°C for about 2 days before the experiment. For summer experiments, the cultures were acclimated for 6 h at room temperature before preparing the test mixtures.

The experimental design was the same for both experiments. Older copepodites (CIV-V, females; 60–65 ind. species^–1^) were incubated individually in 6-well microplates filled with 0.2-μm filtered seawater (5 mL) and offered a food mixture containing 35% of *N. spumigena* and 65% of *R. salina* (by carbon) at a total density of 8 × 10^6^ cell mL^–1^. This ratio of the food mixture was chosen to represent the maximal cyanobacteria contribution to the summer phytoplankton assemblage in the area (Hogfors et al., 2014; Motwani et al., 2017). The control animals (45–50 ind. species^–1^) received labeled *R. salina* at the same density as the sole food. No treatment with 100% *Nodularia* diet was possible because of the high (∼90%) mortality of the copepods on the pure cyanobacteria diet; also, such a diet would not be ecologically relevant for these copepods (Hogfors et al., 2014). The microplates were placed in a temperature-controlled room (17 and 6°C in the summer and winter experiments, respectively) with a dim-light (12L:12D light: dark cycle) and incubated for 24 h. At the termination of the experiment, the copepods were assigned to two groups designated for (1) stable isotope analysis (SIA: 3–4 replicate samples, 10–12 ind. sample^–1^); and (2) analysis of RNA:DNA ratio, a proxy for growth (7–10 replicate samples, 1 ind. sample^–1^). The specimens designated for RNA: DNA measurements were preserved with RNA*later* by species/treatment groups and stored at -20°C until analysis. For SIA sample preparation, the experimental animals were first subjected to purging the gut contents to eliminate the isotope signal due to the food bolus. The copepods designated for SIA were transferred with a Pasteur pipette to a new microplate filled with the new media and the same type of the feeding mixture as in the experiment but with unlabeled foods. After 4 h, the copepods were rinsed with distilled water and used for SIA samples.

### Detection and Quantification of *mlrA* Gene

Using a 10% Chelex buffer (Giraffa et al., 2000), genomic DNA was extracted from the individual copepods preserved in RNA*later* (9–10 ind. sample^–1^). The animals were homogenized in 200 μl of 10% Chelex and boiled at 100°C for 30 min. Each sample was centrifuged at 12,000 g for 2 min, and the supernatant was transferred to a clean tube and kept at −20°C. The DNA concentration and the purity were determined using Nanophotometer (Implen). As a standard, a synthetic DNA oligonucleotide (Vermeulen et al., 2009) comprising the 120-bp target sequence was constructed using *Sphingosinicella microcystinivorans* strain Y2 sequence (Maruyama et al., 2006); GenBank accession number AB114203.1 and synthesized by Applied Biosystems (Cheshire, United Kingdom). The standards were applied in five-step 10-fold serial dilutions, 2 × 10^6^ to 2 × 10^2^ apparent copies of target DNA per reaction, with the *mlrA* primer and probe set according to Hoefel et al. (2009): qmlrAf: 5′-AGCCCKGGCCCRCTGC-3′ and qmlrAr: 5′-ATGCCARGCCCACCACAT-3′ and TaqMan probe qmlrA-tm: 5′-FAM- TGCCSCAGCTSCTCAAGAAGTTTG-BHQ1-3′. The efficiency and determination coefficient (R^2^) of the standard curve were 95–102% and 0.98–0.99, respectively.

All reactions were performed in duplicates using a Real-Time PCR machine (StepOne, Applied Biosystems). The 25-μL reaction volume contained 1× QuantiTech Probe PCR Master Mix (QIAGEN), 0.4 μM of each primer, 0.2 μM of each probe and 1 μL of either a DNA standard or a sample. Thermal cycling conditions for *mlrA* gene amplification were set according to the manufacturer’s instructions and with annealing/extension at 62°C for 1 min (Hoefel et al., 2009). Spiking and recovery tests were conducted to evaluate differences in matrix effects between the species.

Quantification of gene copies per sample was calculated using the standard curve and the Ct values calculated by the StepOne software. In the test samples, *mlrA* copy number per reaction was estimated using the molecular weight of the standard:

$$\mathrm{CN}=\frac{A\left(\times \mathrm{DNA}\right)}{\mathrm{MW}}$$

where *CN* is number of copies (μL^–1^), *A* is 6 × 10^23^ is the gene copies mol^–1^ (the Avogadro constant), *DNA* is DNA concentration (g μL^–1^), and *MW*– molecular weight of the amplicon 37,012 g mol^–1^ provided by the manufacturer of the synthetic standard (Rodrigo et al., 1997). The calculated CN values were further adjusted for the number of copepods per sample (9 or 10 ind.) to calculate *mlrA* copy number per individual.

### SIA

The copepods collected at the start and the termination of the feeding experiment were transferred to tin capsules, dried at 60°C for 48 h, weighed (∼0.2–0.3 mg dry mass sample^–1^), and stored in a desiccator until shipped to the analytical facilities. The samples were analyzed at the Center for Physical Science and Technology, Vilnius, Lithuania. The relative abundance of stable carbon and nitrogen isotopes were measured using a Flash EA 1112 Series Elemental Analyzer connected via a Conflo III to a DeltaV Advantage isotope ratio mass spectrometer (all Thermo Finnigan, Bremen, Germany). Ratios of ^14^N:^15^N and ^12^C:^13^C are expressed relative to the international standards, atmospheric air (N) and Pee Dee Belemnite (C). Internal reference (pike muscle tissue) was analyzed every 10 samples. Overall analytical precision was 0.15 ‰ for δ^15^N and 0.10 ‰ for δ^13^C.

### RNA:DNA Ratio

This ratio was used as a proxy for overall metabolic activity in the test copepods (Höök et al., 2008; Holmborn et al., 2009) representing the growth response to the experimental diets. The nucleic acid concentrations were measured in the RNA*later*-preserved copepods collected in the field survey (Table 1) and upon the termination of the feeding experiments. For each individual, RNA and DNA concentrations (mg ind.^–1^) were measured using microplate fluorometric high-range RiboGreen (Molecular Probes, Inc. Eugene, OR) assay (Gorokhova and Kyle, 2002) optimized for *Eurytemora affinis* of similar size (Höök et al., 2008). All test samples, standards, and the negative controls were measured in duplicates using FLUOstar Optima microplate reader in black solid flat-bottom microplates (Greiner Bio-One GmbH) at excitation/emission wavelengths of 485/590 nm (0.2 s well^–1^, 10 measurements well^–1^). The DNA: RNA standard curve slope ratio was 1.59.

### Data Analysis and Statistics

Carbon and nitrogen incorporation indices (CINC and NINC, respectively) were calculated as:

$$X=\frac{\left[X_{t}-X_{0}\right]}{\left[X_{\mathrm{diet}}-X_{0}\right]}$$

where *X*_*t*_ is the δ-value for element X (carbon or nitrogen) at time *t* (end of the experiment), *X*_0_ is the starting δ-value and *X*_*diet*_ is the δ-value for the experimental diet (Paulet et al., 2006). CINC and NINC integrate growth and turnover-processes during the incubation time. When isotopic equilibrium cannot be reached during the experiment as was the case in our short-term incubations, the analysis of carbon and nitrogen incorporation indices is useful in identifying growth activity and metabolic costs reflecting the physiological status (Ek et al., 2016). The CINC and NINC values were used as a proxy for the uptake of *Nodularia spumigena* and *Rhodomonas salina*, respectively.

Generalized linear model (GLM) analysis with normal error structure and log link as implemented in STATISTICA 8.0 (StatSoft, 1984–2007) was used to evaluate the relationships between the *mlrA* gene abundance and (1) carbon biomass of hepatotoxin-producing cyanobacteria (*Nodularia spumigena* and *Dolichospermum* sp.) at the time of collection, and (2) uptake of *N. spumigena* and *R. salina*, using CINC and NINC, respectively. Moreover, GLMs were used to evaluate relative importance of *mlrA* gene abundance, grazing rate and assimilation as predictors for variation in the RNA:DNA ratio. *Season* and *Species* × *Season* interaction term were included in all regressions. The Wald statistic was used to check the significance of the regression coefficient for each parameter, a likelihood ratio test was used to evaluate the statistical significance of including or not including each parameter and model goodness of fit was checked using deviance and Pearson χ^2^ statistics. Residual plots for each model were assessed visually to exclude remaining un-attributed structure indicative of a poor model fit. Akaike Information Criterion (AIC) was used to compare alternative models and to select the most parsimonious model (Burnham and Anderson, 2004). In all statistical analyses, the significance level was α = 0.05; if not specified otherwise, the data are presented as a mean and standard deviation.

## Results

### Field Survey

**FIGURE 1:**
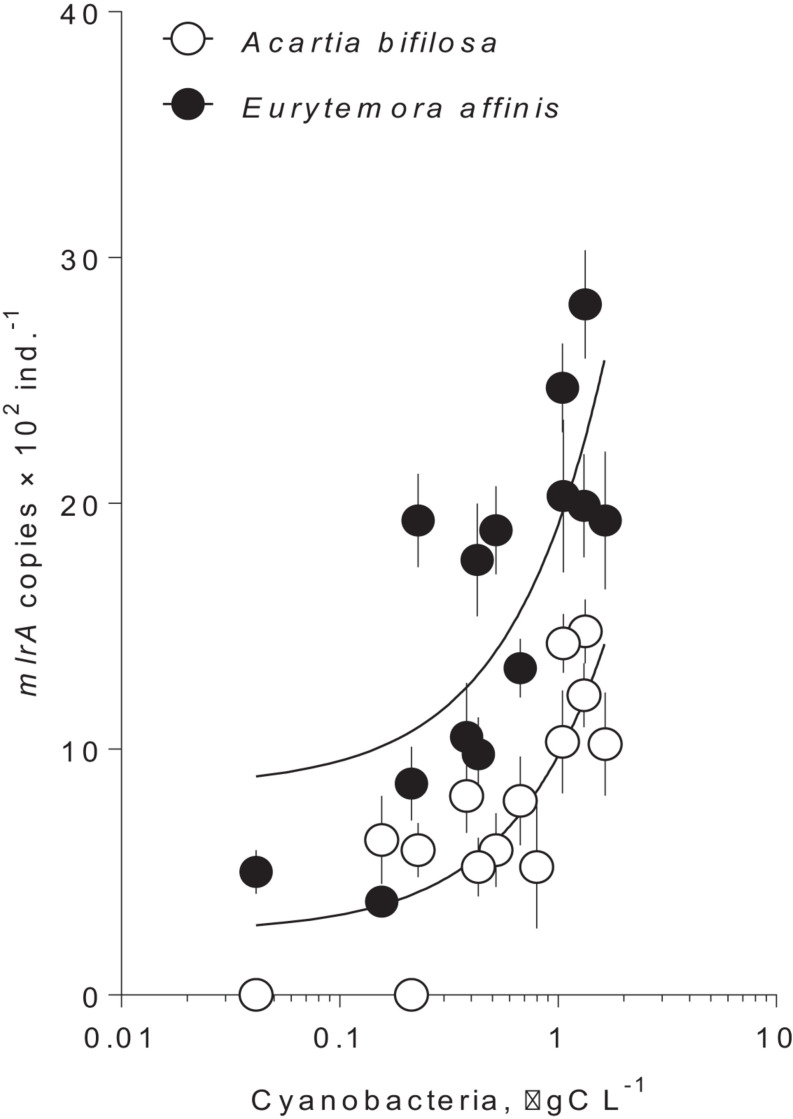
Occurrence of *mlrA* gene in microbiome of the field collected copepods *Acartia bifilosa* and *Eurytemora affinis* as a function of hepatotoxic cyanobacteria biomass at the time of collection during summer (Table 1; 2009–2011). The winter values are not shown because of the zero values for the cyanobacterial biomass and significantly lower values for the *mlrA* gene abundance. Cyanobacteria that were included are *Nodularia spumigena* and *Dolichospermum* sp. The data are shown as mean and standard deviation based on 2–4 replicate samples. The lines are the linear regressions in the semi-logarithmic scale; see Supplementary Table S2, for the regression details.

In summer, the *mlrA* gene was detected in >90% of the copepod samples varying from below quantification limit to1,500 copies ind.^–1^ in *Acartia* and from 400 to 3,000 copies ind.^–1^ in *Eurytemora* (Figure 1 and Supplementary Table S1). For each species, there was a significant relationship between the *mlrA* gene abundance and the ambient carbon biomass of the cyanobacteria capable of nodularin or microcystin production (Figure 1 and Supplementary Table S2). Moreover, the *mlrA* gene abundance was consistently higher in *Eurytemora* [slope: *F*_(1,24)_ = 0.72, *p* > 0.40; intercept: *F*_(1,25)_ = 29.32, *p* < 0.0001; Supplementary Table S2]. In winter, the *mlrA* gene abundance varied from 210 to 400 copies ind.^–1^ in *Acartia* and from 980 to 1,300 copies ind.^–1^ in *Eurytemora* and was significantly lower than in summer for both species (Unpaired *t*-test with Welch’s correction for unequal variances; *Acartia*: *t*_(19.99)_ = 3.984, *p* < 0.0007, *F* test to compare variances: *F*_(17, 3)_ = 24.05, *p* < 0.02; and *Eurytemora*: *t*_(16.71)_ = 3.317, *p* < 0.004; *F*-test to compare variances: *F*_(17, 2)_ = 21.40, *p* < 0.09).

### Feeding Experiments

The survival in all species/treatment groups was >90%, with no apparent variation related to the treatment or the species. Slightly lower survival was observed in the winter experiment (Supplementary Figure S2), which was most likely the result of the temperature stress during the handling of the animals from the winter populations. In both species, some individuals molted during the experiment and became mature females. For both species, the body mass was significantly larger in winter than in summer, with no significant interaction and difference between the species within a season (Supplementary Table S3 and Supplementary Figure S3). As no significant difference in body mass between the species was found, the *mlrA* copy number was expressed on the individual basis.

**FIGURE 2:**
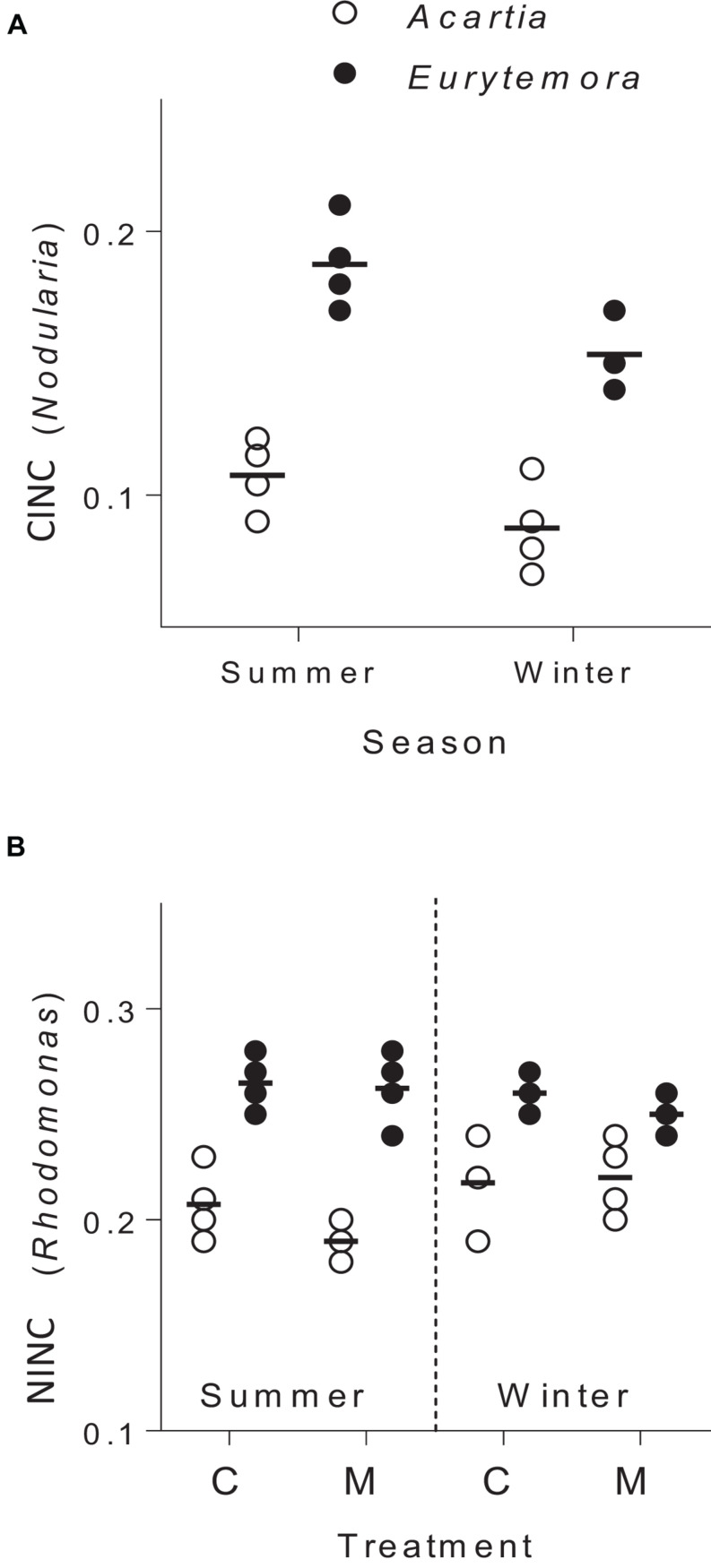
Incorporation of *Nodularia spumigena* (**A**; carbon incorporation index, CINC) and *Rhodomonas salina* (**B**; nitrogen incorporation index, NINC) by *Acartia bifilosa* and *Eurytemora affinis* in the feeding experiments conducted in summer (August) and winter (February). The animals were exposed to either control food (C; 100% *R. salina*) or a mixture containing 35% of ^13^C-labeled *N. spumigena* and 65% of ^15^N-labeled *R. salina* (M). Each treatment has four replicates per species, except the winter trials with *Eurytemora affinis* where three replicates were used; the horizontal line indicates the mean value for a treatment. See Supplementary Tables S5, S6 for the statistical comparisons.

The copepods in the food mixtures and the controls were feeding during the trial, as evidenced by the stable isotope uptake (Figure 2). The assimilation of *N. spumigena* was significantly higher in *Eurytemora* compared to *Acartia*, as indicated by the higher values of CINC in the former species (Supplementary Table S4 and Figure 2A). The assimilation of *R. salina* was also higher in *Eurytemora* (Supplementary Table S5A and Figure 2B). Within a species, no diet effect on the *R. salina* uptake was found, as evidenced by the similar NINC values between the control and the mixed diet (Supplementary Table S6).

**FIGURE 3:**
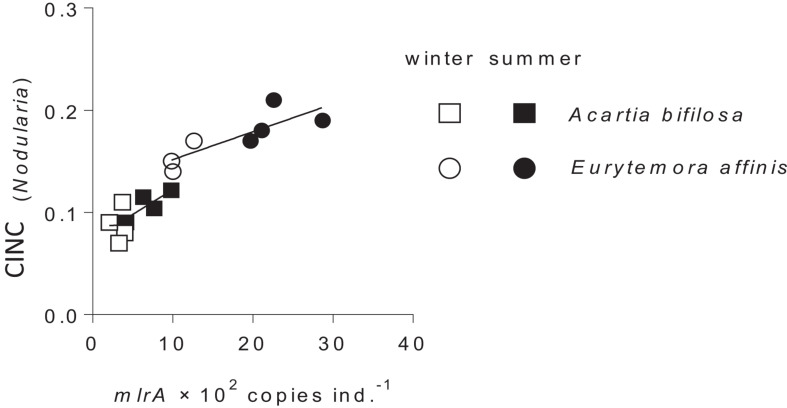
Incorporation of *Nodularia spumigena* reflected in carbon incorporation index (CINC) by *Acartia bifilosa* and *Eurytemora affinis* in the feeding experiments conducted in summer (August) and winter (February) as a function of *mlrA* gene abundance in the microbiome of the copepods sampled from the same population as the experimental animals. The animals were exposed to a food mixture containing 35% of ^13^C-labeled *N. spumigena* (by carbon mass). Both *species* and *season* effects are significant; see Table 2 and Supplementary Table S4 for statistical comparisons.

Following the gut clearance procedure, the *mlrA* gene was detected in all copepod samples, with significantly higher values in *Eurytemora* than in *Acartia* and significant positive relationships with the CINC values (Figure 3 and Table 2). Moreover, the variation in the *mlrA* gene copies ind.^–1^ measured at the start of the incubation was a better predictor for the uptake of *N. spumigena* measured as CINC values than *Season* (Supplementary Table S7). The uptake of *R. salina* measured as NINC was not related to the *mlrA* gene occurrence (Supplementary Table S5B).

**TABLE 2:** GLM output for the carbon incorporation index (CINC) representing uptake of ^13^C-labeled *Nodularia spumigena* in the feeding experiment as a function of the *mlrA* gene abundance in the microbiome of the field-collected *Acartia bifilosa* and *Eurytemora affinis* used in the experiment; see Figure 2 for visualization of the data.

	**Reference**	**Estimate**	**Wald stat.**	***p-*value**
Intercept		−2.236	1354.2	<0.0001
*mlrA*		0.016	14.9	0.0013
*Species*	*Acartia*	−0.175	17.5	0.0001
*Species* × *mlrA*		0.001	1.4	0.2335

**FIGURE 4:**
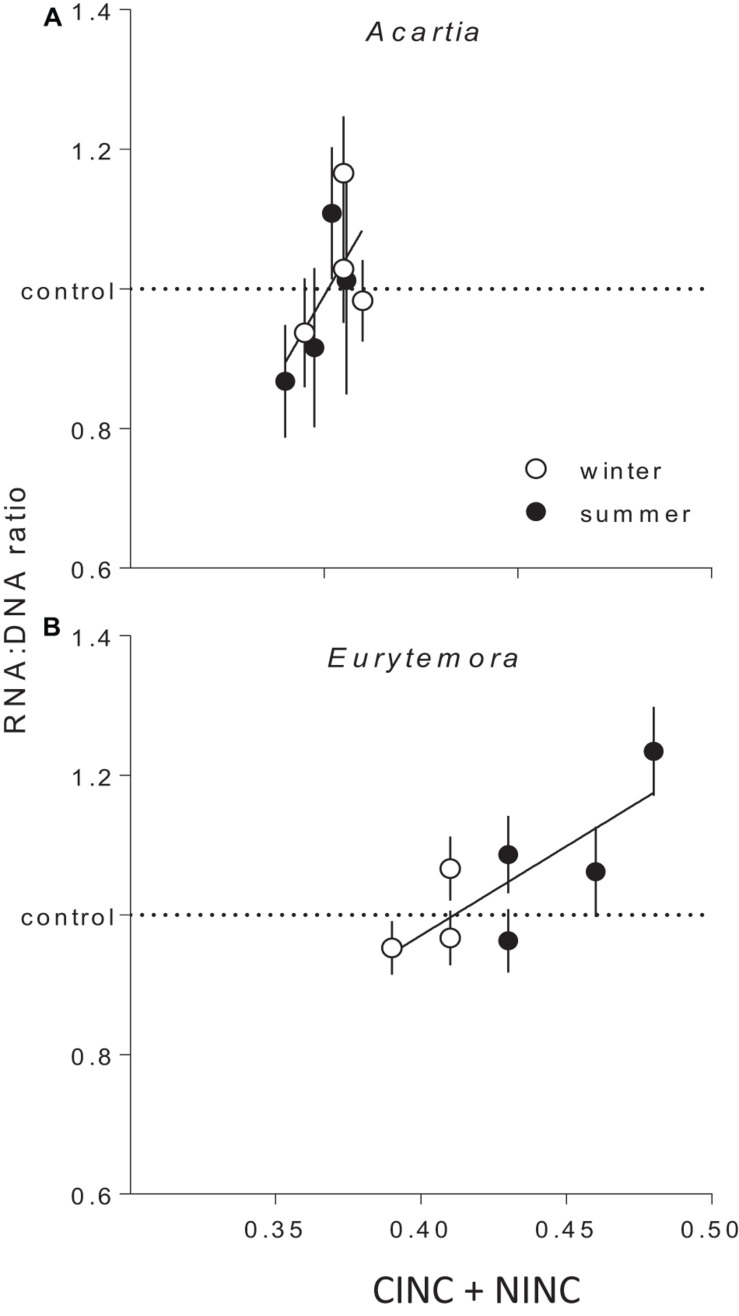
The RNA:DNA ratio in *Acartia bifilosa* **(A)** and *Eurytemora affinis* **(B)** in relation to the total uptake of cyanobacteria (*Nodularia spumigena*; measured as carbon incorporation index CINC) and non-cyanobacterial food (*Rhodomonas salina*; measured as nitrogen incorporation index NINC) in the feeding experiments with mixed diets conducted in summer and winter. To account for the differences in the RNA:DNA ratio between the seasons within a species (Supplementary Figures S4, S5), all values were normalized to the controls, i.e., the RNA:DNA ratios in the same species exposed to *Rhodomonas salina* as a sole food (dashed line). Each treatment has four replicates per species, except winter trials with *Eurytemora affinis* where three replicates were used. As a replicate, we considered a set of animals sampled from a particular location on a particular date and exposed to the same experimental conditions within a treatment. Data are shown as mean values (*n* = 9 or 10) with SD; the regressions are based on the mean values. See Supplementary Table S5 for the regression parameters.

The copepod RNA:DNA ratio was significantly different between the species and seasons, with higher values observed in *Eurytemora* than in *Acartia* and in winter than in summer (Supplementary Figures S4, S5). These differences were expected as (1) *Acartia* has higher DNA% compared to *Eurytemora*, and (2) RNA:DNA ratio is higher at lower temperature (Holmborn and Gorokhova, 2007). As the mechanisms behind these differences were not related to our research questions, the RNA:DNA ratios observed in the feeding experiments with mixed diets were normalized to those in the controls fed *Rhodomonas* as a sole food. In both species, the normalized RNA:DNA ratio was positively related to the total food uptake as reflected by the CINC+NINC values, the effect being significant for *Eurytemora* (*R*^2^ = 0.63, *p* < 0.03) and marginally significant for *Acartia* (*R*^2^ = 0.40, *p* > 0.09; Figure 4 and Supplementary Table S8). When CINC and NINC were used as sole predictors, the relationships were not significant (Supplementary Table S9).

## Discussion

In the microbiome of *Acartia bifilosa* and *Eurytemora affinis*, bacterial genes *mlrA* are ubiquitously present, with their occurrence being significantly higher in the latter species. As hypothesized, the abundance of *mlrA* genes in the copepod microbiome increased during summer, in concert with the stocks of hepatotoxic cyanobacteria in the water. Furthermore, the experimental exposure of the copepods to *Nodularia spumigena* confirmed that the *mlrA* gene abundance was the best predictor of the uptake of the cyanobacteria carbon by the consumers, thus supporting the functional involvement of the *mlrA*-carriers to the copepod capacity to handle the cyanobacteria in the diet. As these genes are involved in the microcystin/nodularin degradation pathway, their occurrence in the copepod microbiome suggests that bacteria carrying *mlrA* can contribute to biodegradation of these toxins ingested by the host and thus facilitate their nutrition during the bloom events.

The evidence is accumulating that exposure to cyanobacteria can shift zooplankton communities toward better-adapted species, select for more tolerant genotypes within a species, and induce traits within the lifetime of individual zooplankters (Ger et al., 2016). Recent experimental studies have demonstrated that the gut microbiota transplant from a microcystin-tolerant clone can increase tolerance to *Microcystis*-rich diet in *Daphnia magna* (Macke et al., 2017). Therefore, the ingestion of fecal particles and, in the case of copepods, fecal pellets containing gut microbiota, including taxa capable of cyanotoxin biodegradation, by zooplankters (within and between species and populations) and benthic animals could enrich these consumers with the microflora of cyanotoxin-tolerant phenotypes. Also, ambient bacteria, both free-living and occurring in biofilms, can enter the consumer gut and become a part of the resident microbiota (Hansen and Bech, 1996).

Known degraders of microcystin and nodularin are present in the Baltic cyanobacteria blooms (Pinhassi and Hagström, 2000; Tuomainen et al., 2006). In particular, *Sphingomonas* sp., β-proteobacteria (*Rhodoferax* sp.) as well as *Pseudomonas* sp., all putative cyanotoxin biodegraders, are very abundant during summer in the Baltic proper, concomitant with the blooms (Pinhassi and Hagström, 2000; Ininbergs et al., 2015; Hu et al., 2016). In line with this, the abundance of *mlrA* gene in the copepods was positively related to the ambient biomass of the known nodularin and microcystin producers (*Nodularia spumigena* and *Dolichospermum* spp., respectively). In winter, however, when none of these cyanobacteria were present in plankton, the copepod samples were still positive for the *mlrA* gene, indicating that carriers of this gene are present in the microbiota all year round, increasing in concert with cyanobacteria, which may represent an adaptation to the resource availability. In other systems experiencing regular blooms of hepatotoxic cyanobacteria, seasonal variability in *mlrA* gene abundance has also been reported. For example, in some freshwater lakes, the abundance of *Microcystis* spp. co-varied with that of the microcystin-degrading bacteria (Zhu et al., 2014; Lezcano et al., 2017). Moreover, Lezcano et al. (2017), demonstrated that although the *mlrA* gene is always present in the water, its abundance followed the abundance of the *mcyE* gene, which is a gene involved in microcystin biosynthesis. Predicting how ambient bacterioplankton and horizontal transfer of the resistance may affect zooplankton microbiome and adaptation to cyanotoxins requires more experimental studies. In particular, the activity of the microbiome *mlrA*-carriers in nodularin/microcystin degradation during digestion, which may also vary among species, need to be compared using transcriptional expression analysis.

Of the two copepod species, *E. affinis* harbored more *mlrA* copies than *A. bifilosa*; this holds for both summer and winter populations (Figures 1, 2). Moreover, when exposed to *Nodularia*-rich diet, *E. affinis* was consistently superior with regard to food assimilation and growth potential as suggested by the RNA:DNA ratio response (Figures 2–4). These findings provide a mechanistic explanation of the reported differences in the adaptation strategies to the cyanobacteria blooms between the two copepod species in the Northern Baltic Proper. In *E. affinis* populations co-occurring with cyanobacterial blooms, positive effects of *Nodularia* ingestion on growth, reproductive output, and naupliar survival have been reported (Hogfors et al., 2014; Motwani et al., 2017), indicating that all ontogenetic stages of this species are well-adapted to the cyanotoxins and can efficiently utilize cyanobacteria. By contrast, ingestion of *Nodularia* by *A. bifilosa* may adversely affects egg production (Engström-Öst et al., 2015), although positive effects on RNA:DNA ratio, egg viability and nauplial abundance have also been reported for this species (Hogfors et al., 2014). *Acartia* species can switch to ambush feeding and thus are more selective toward large motile prey (Jonsson and Tiselius, 1990), avoiding cyanobacteria, and hence might be less dependent on the cyanotoxin biodegraders in the gut. However, in both species, the capacity to digest and assimilate *Nodularia* was positively related to the *mlrA* gene occurrence (Figure 3). Moreover, the growth potential assessed as RNA:DNA ratio was significantly positively related to the total uptake of food comprised of cyanobacteria and *R. salina* (Figure 4). Thus, the microbiome was responding to the ingested dietary toxins by increasing the *mlrA* gene abundance, and some adaptation can be expected even in less tolerant species, such as *Acartia bifilosa.*

Although both species were similarly-sized, implying low interspecific variability in the gut size within a season, and sampled from the same communities, implying that they were exposed to the same ambient bacterioplankton, the abundance of *mlrA* genes between the copepods differed significantly. As copepods, similar to other filtrators, acquire much of their gut microflora via ingestion of ambient bacterioplankton (Tang et al., 2010), one reason for this differential abundance could be a higher intake rate of bacteria carrying the *mlrA* gene by *Eurytemora* that has a higher feeding on the bacteria-sized prey compared to *Acartia bifilosa* (Motwani and Gorokhova, 2013). Thus, the differential bacterivory may explain some of the interspecific variability in the *mlrA* gene abundance observed in the field survey. However, the experimental animals were pre-incubated with axenic algae in 0.2-μm filtered seawater, and their experimental foods were grown in sterile media. Treated in this way, *Eurytemora* hosted more *mlrA* copies than *Acartia*, which suggests that the carriers of this gene were a part of the resident microbiome. Therefore, the differences in the *mlrA* load between the species in the experiment indicate that the genetic background of the host may also control gut microbiota by sorting out the acquisition and establishment of the bacterial taxa in the gut, which suggest differences in the evolutionary potential between *E. affinis* and *A. bifilosa* to prosper in a cyanobacteria-dominated ecosystem. In turn, the frequency and magnitude of the cyanobacterial blooms would present a selection pressure on grazer populations (Drugǎ et al., 2016).

The non-toxic food was assimilated equally well regardless of the diet, although we expected that *Rhodomonas* would be assimilated less efficiently in the presence of *Nodularia* due to the production of the protease inhibitors by the latter. These inhibitors have been shown to inhibit trypsins and chemotrypsins, i.e., the key groups of digestive proteases in zooplankters (Agrawal et al., 2005; Schwarzenberger et al., 2010). Although we did not measure the protease levels and activity, the uptake of ^15^N indicates that both copepods were digesting *Rhodomonas* at constant efficiency, both in controls and mixed diets (Supplementary Figure S5). Notably, the assimilation of *Rhodomonas* was also higher in *Eurytemora* than in *Acartia*, suggesting either higher ingestion or higher assimilation efficiency in the former, which may also be related to the overall differences in the microbiome between the species, including the relative abundance of *mlrA* genes. For example, Sphingomonadales, the well-known *mlrA*-carriers, have a diverse metabolic capacity and utilize various organic substances, which may affect the overall metabolic capacity of their hosts. Also, in addition to the microcystin/nodularin neutralization, members of the order Sphingomonadales may provide a broader defense from other toxins and bioactive compounds (Festa et al., 2017).

The diversity of gut microbiome in animal hosts and their role in shaping the host physiology and ecological adaptations in arthropods is gaining attention (Degli Esposti and Martinez Romero, 2017). Our knowledge of such interactions in planktonic communities is insufficient, and no cyanotoxin-degrading gut symbiont has been identified in aquatic consumers so far. However, studies on terrestrial arthropods provide convincing evidence that their symbionts facilitate grazing on plants producing bioactive compounds. For example, the mountain pine beetle colonizes conifers that synthesize terpenoids, which are toxic to beetles, with the help of a microbiome enriched with genes involved in terpene degradation (Adams et al., 2013) and metabolization of monoterpenes and diterpene acids (Boone et al., 2013). Also, gut bacteria of gypsy moth reduce the concentrations of phenolic glycosides that inhibit gypsy moth growth (Mason et al., 2014). Our results provide empirical evidence that biodegrading bacteria in the microbiome of copepods play an active role in providing their host with tolerance to toxic cyanobacteria when frequently present in the food diet. Both host genotype and pool of the biodegrading bacteria in the environment determine zooplankton capacity to acquire these bacteria as an ecological adaptation. Therefore, research questions regarding adaptation and competition processes in plankton communities should be addressed at the holobiont level.

## Data Availability Statement

The raw data supporting the conclusions of this article will be made available by the authors, without undue reservation.

## Author Contributions

EG and RE-S conceived the study, undertook the molecular analyses, and wrote the manuscript. EG and ML conducted the experiments and collected the samples. AG undertook the SIA. EG undertook the data analysis. All authors commented on the manuscript and reviewed the final draft.

## Conflict of Interest

The authors declare that the research was conducted in the absence of any commercial or financial relationships that could be construed as a potential conflict of interest.

## Footnotes

1 www.smhi.se

2 http://www2.ecology.su.se/dbhfj/index.htm
