# Supplementary material for: How Copepods Can Eat Toxins Without Getting Sick: Gut Bacteria Help Zooplankton to Feed in Cyanobacteria Blooms
Source: Front Microbiol. 2021 Jan 12;11:589816. doi: 10.3389/fmicb.2020.589816 (PMC7835405; doi:10.3389/fmicb.2020.589816)

Supplementary Information

How copepods can eat toxins without getting sick: Gut bacteria help zooplankton to feed in cyanobacteria blooms

Elena Gorokhova[1*], Rehab El-Shehawy[1], Maiju Lehtiniemi[2], and Andrius Garbaras[3]

[1]Department of Environmental Science and Analytical Chemistry, Stockholm University, SE-106 91 Stockholm, Sweden

[2]Marine Research Centre, Finnish Environment Institute (SYKE), P.O. Box 140, 00251 Helsinki, Finland

[3]Mass Spectrometry Laboratory, Center for Physical Science and Technology, Savanoriu 231, LT-02300 Vilnius, Lithuania

*Correspondence author: elena.gorokhova@aces.su.se

Table S1. Summary of data on the *mlrA* gene abundance (mean and SD) in the field-collected copepods *Acartia bifilosa* and *Eurytemora affinis* and the carbon biomass of cyanobacteria occurring in concert with the copepod sampling. Number of samples (*n*) corresponds to the number of replicate samples (10 ind. sample^{-1}) selected from a single net tow on each sampling occasion. As hepatotoxin producers, *Nodularia spumigena* and *Dolichospermum* sp. were included in the cyanobacteria biomass estimates. All stations are located in the Western Gotland Basin; see Table 1 for geographic coordinates and Figure 1 for the graphical presentation.

Station	Date	Biomass of hepatotoxin producers, μg C L^{-1}	*mlrA* copies × 10^2 ind.$^{-1}$					
			A. bifilosa			*E. affinis*		
			mean	SD	*n*	mean	SD	n
B1	2010-06-21	1.40	8.1	1.5	3	10.5	2.2	3
	2010-07-06	2.30	5.9	1.5	3	18.9	1.8	3
	2010-08-03	3.70	7.9	1.8	3	13.3	1.2	3
	2010-08-17	19.61	12.2	1.3	3	19.9	2.1	2
BY31	2011-06-21	0.64	0	0	2	8.6	1.5	3
	2011-07-06	10.36	14.3	1.2	3	20.3	3.1	3
	2011-07-18	10.16	10.3	2.1	2	24.7	1.8	3
	2011-07-20	20.39	14.8	1.3	3	28.1	2.2	3
	2011-08-02[a]	5.28	5.2	2.5	3			
	2011-08-03	43.26	10.2	2.1	3	19.3	2.8	3
	2011-08-16	1.70	5.2	1.2	2	9.8	1.5	2
	2011-08-17[b]	1.67				17.7	2.3	3
H4	2009-07-14	0.09	0	0	3	5	0.9	3
F69	2016-02-11	0	3.7	1.1	2	10.1	1.9	3
F69	2016-02-16[a]	0	2.1	0.8	2			
BY32	2016-02-13	0	4.0	1.2	3	9.8	1.5	4
AALTOPI	2016-02-15	0	3.3	0.7	3	12.7	0.9	3

[a] No *Eurytemora affinis* samples were available for this date

[b] No *Acartia bifilosa* samples were available for this date

Table S2. Regression details for the relationship between the *mlrA* gene abundance in the microbiome of the field-collected copepods *Acartia bifilosa* and *Eurytemora affinis* and the biomass of the hepatotoxic cyanobacteria at the time of the copepod collection (stations B1, BY31, and H4; 2009-2011) during summer. The mean values for each sampling occasion were used in the least square regression analysis; see Fig. 1 for the data visualization.

**Regression parameters**	*Acartia bifilosa*	*Eurytemora affinis*
Slope	7.37 ± 1.54	10.66 ± 2.74
Intercept	2.54 ± 1.32	8.45 ± 2.29
95% confidence interval for slope	3.84 to 10.56	4.69 to 16.62
R^2	0.64	0.56
$F_{1,12}$	21.78	15.14
p value	0.0005	0.002

Table S3. ANOVA results for comparison of the individual body size (dry weight, copepodites CV, females) between the species (*Acartia bifilosa* vs. *Eurytemora affinis*) and seasons (summer vs. winter). See Fig. S2 for visualization of the data.

**Parameters**	**SS**	**MS**	$\mathbf{F_{1,11}}$	***p***
Season × *Species*	0.020	0.01963	1.923	0.19
Season	2.81	2.813	275.6	< 0.0001
Species	0.0085	0.0085	0.8305	0.38
Residual	0.1123	0.0102		

Table S4. GLM output of the feeding experiment data showing significant effects of *Species* and *Season* on the carbon incorporation index (CINC) that represents uptake of ^{13}C-labeled *Nodularia spumigena* by the copepods. The model indicates that *Acaria bifilosa* has significantly lower ^{13}C incorporation compared to *Eurytemora affinis* regardless of the season. See Fig. 2 for visualization of the data.

**Parameters**	**Level of Effect**	**Estimate**	**Wald Stat.**	***p* value**
Intercept		-2.053	4803.452	<0.0000
Season	summer	0.102	11.875	0.0006
Species	*Acartia*	-0.278	88.646	<0.0000
Season × *Species*		0.002	0.003	0.9590

Table S5. GLM output of the feeding experiment showing (A) no effect of *Season* but a significant effect of *Species* on the nitrogen incorporation index (NINC) representing uptake of ^{15}N-labeled *Rhodomonas salina* by the copepods in the control treatment; and (B) no effect of *mlrA* on NINC in the copepods exposed to the mixed diet. Both models indicate that *Acaria bifilosa* has significantly lower ^{15}N incorporation compared to *Eurytemora affinis* regardless of the season and the occurrence of the *mlrA*-carrying bacteria in their microbiome.

(A)

**Parameters**	**Reference**	**Estimate**	**Wald Stat.**	***p***
Intercept		-1.443	8958.473	0.0000
Species	*Acartia*	-0.106	48.110	0.0000
Season	summer	-0.007	0.211	0.6459
Species × *Season*	1	-0.016	1.175	0.2783

(B)

**Parameters**	**Reference**	**Estimate**	**Wald Stat.**	***p***
Intercept		-1.462	1032.7	0.0000
mlrA		-0.001	0.1	0.8294
species	*Acartia*	-0.118	15.7	0.0001

Table S6. Differences between NINC values measured in different experiments (summer vs. winter) and exposure conditions (control vs. food mixture) analyzed by a Holm-Sidak multiple comparison test for *Acartia bifilosa* and *Eurytemora affinis*.

**Multiple comparisons**	**Mean Diff.**	**95% CI of diff.**	**Summary**
Acartia bifilosa			
summer (control) vs. summer (mixture)	0.0175	-0.0137 to 0.0487	ns
summer (control) vs. winter (control)	-0.0100	-0.0412 to 0.0212	ns
summer (mixture) vs. winter (mixture)	-0.0300	-0.0612 to 0.0012	ns
winter (control) vs. winter (mixture)	-0.0025	-0.0337 to 0.0287	ns
Eurytemora affinis			
summer (control) vs. summer (mixture)	0.0025	-0.0287 to 0.0337	ns
summer (control) vs. winter (control)	0.0050	-0.0287 to 0.0387	ns
summer (mixture) vs. winter (mixture)	0.0125	-0.0212 to 0.0462	ns
winter (control) vs. winter (mixture)	0.0100	-0.0260 to 0.0460	ns

Table S7. Comparison of the best-fit models describing effects of the *mlrA* gene abundance, *Season* and *Species* on the uptake of *Nodularia spumigena* measured by CINC in the test copepod species. For each model, the degrees of freedom (df), the Akaike Information Criterion (AIC), the likelihood ratio and the model *p* value are shown.

	**Var. 1**	**Var. 2**	**df**	**AIC**	**Likelihood Ratio, Chi^2**	***p* value**
**1**	*mlrA*	*Species*	2	-80.35	33.990	<0.0001
**2**	*Season*	*Species*	2	-78.28	33.913	<0.0001

Table S8. Regression details for the relationship between the normalized RNA:DNA ratio in the copepods (*Acartia bifilosa* and *Eurytemora affinis*) and total food uptake, including *Nodularia spumigena* and *Rhodomonas salina* in the feeding experiments; CINC and NINC were used as proxies for *Nodularia* and *Rhodomonas* uptake, respectively. The mean values for each replicate were used in the least square regression analysis; see Fig. 4 for the data visualization.

**Regression parameters**	***Acartia bifilosa***	***Eurytemora affinis***
Slope	4.77 ± 2.37	2.55 ± 0.87
Intercept	-0.44 ± 0.72	-0.05 ± 0.37
95% confidence interval for slope	-1.04 to 10.57	0.32 to 4.79
R^2	0.4023	0.6334
F	4.039	8.638
DFn, DFd	1, 6	1, 5
p value	0.09	0.03

Table S9. Regression details for the relationship between the RNA:DNA ratio in the copepods (*Acartia bifilosa* and *Eurytemora affinis*) and uptake of (A) *Nodularia spumigena* in the feeding experiment using CINC and NINC as proxies for *Nodularia* (Fig. S4) and *Rhodomonas* (Fig. S5) uptake, respectively. The mean values for each replicate were used in the least square regression analysis.

**Regression parameters**	***Acartia bifilosa***		***Eurytemora affinis***	
	**summer**	**winter**	**summer**	**winter**
Slope	-0.89 ± 4.85	9.78 ± 9.73	7.38 ± 15.80	5.38 ± 12.12
Intercept	2.22 ± 0.96	2.29 ± 2.13	2.27 ± 4.17	5.60 ± 3.17
95% confidence interval for slope	-12.77 to 10.99	-14.03 to 33.60	-31.28 to 46.06	-28.27 to 39.04
R^2	0.006	0.141	0.035	0.047
F	0.033	1.010	0.218	0.197
DFn, DFd	1, 6	1, 6	1, 6	1, 4
p value	0.86	0.35	0.65	0.67

Figure S1. Nodularin concentration (µg mg dry weight^{-1}) in the ^{13}C-labeled and unlabeled culture of *Nodularia spumigena* (strain AV1) used in the feeding experiments and two-way ANOVA results evaluating the effects of *labeling* and *season*. All cultures were statistically indistinguishable with regard to the intracellular nodularin concentrations.

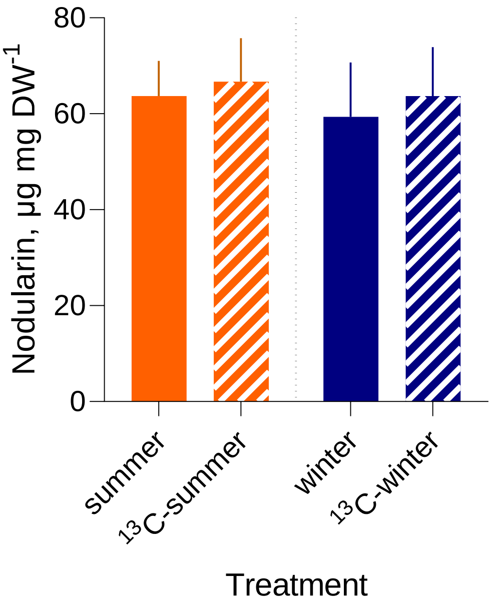

ANOVA table	SS	DF	MS	F (DFn, DFd)	P value
Interaction	1.347	1	1.347	F (1, 8) = 0.01455	0.907
season	40.41	1	40.41	F (1, 8) = 0.4366	0.527
labeling	40.41	1	40.41	F (1, 8) = 0.4366	0.527

Figure S2. Survivorship (%) of *Acartia bifilosa* and *Eurytemora affinis* in the feeding experiments conducted in summer (August) and winter (February). The animals were exposed to either control food (C; 100% *Rhodomonas salina*) or a food mixture (M; 35% of *Nodularia spumigena* and 65% *Rhodomonas salina*).

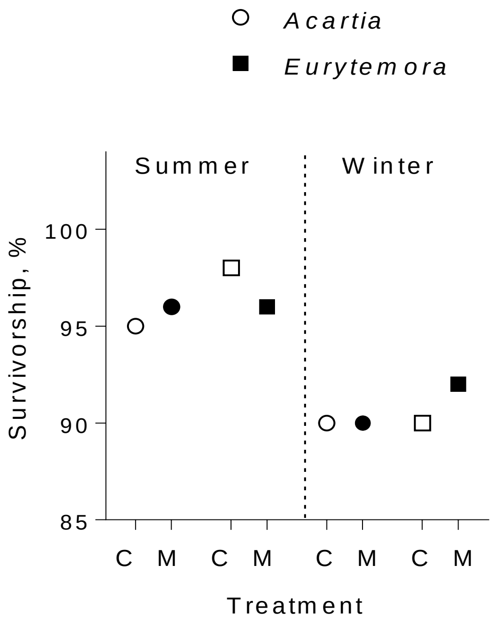

Figure S3. Body size (DW, µg ind^{-1}) of *Acartia bifilosa* and *Eurytemora affinis* used in the feeding experiments conducted in summer (August) and winter (February). See Table S3 for statistical comparisons.

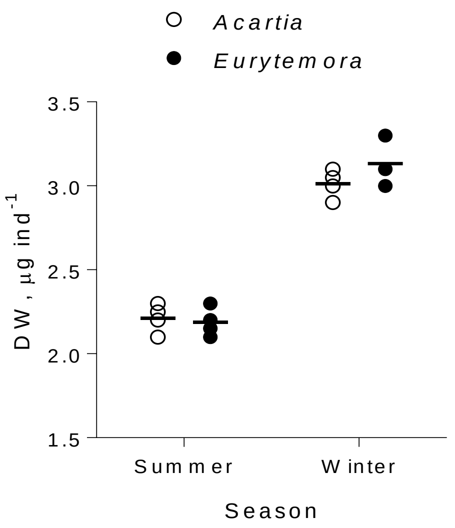

Figure S4. RNA:DNA ratio in *Acartia bifilosa* and *Eurytemora affinis* in relation to the uptake of *Nodularia spumigena* measured as carbon incorporation index (CINC) in the feeding experiments conducted in summer (August) and winter (February). The animals were exposed to a mixture containing 35% of ^{13}C-labeled *N. spumigena* and 65% of *R. salina*. Each treatment has four replicates per species, except winter trials with *Eurytemora affinis* where three replicates were used. As a replicate, we considered a set of animals sampled from a particular location on a particular date and exposed to the same experimental conditions within a treatment. Data are shown as mean values (*n* = 9 or 10) with SD.

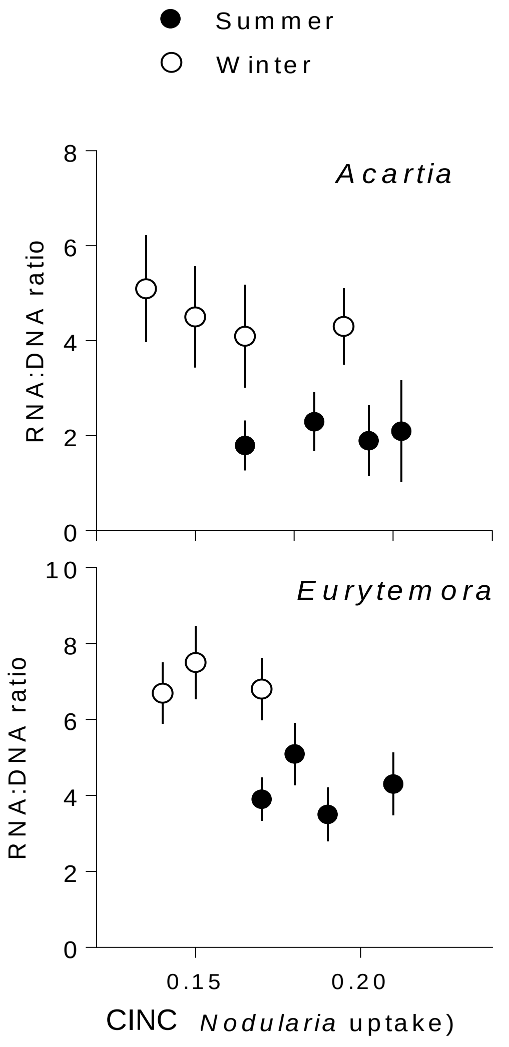

Figure S5. RNA:DNA ratio in *Acartia bifilosa* (A) and *Eurytemora affinis* (B) in relation to the uptake of *Rhodomonas salina* measured as nitrogen incorporation index (NINC) by in the feeding experiments conducted in summer (August) and winter (February). The animals were exposed to either control food (*R. salina*) or a mixture containing 35% of ^{13}C-labeled *N. spumigena* and 65% of ^{15}N-labeled *R. salina*. Each treatment has four replicates per species, except winter trials with *Eurytemora affinis* where three replicates were used. As a replicate, we considered a set of animals sampled from a particular location on a particular date and exposed to the same experimental conditions within a treatment. Data are shown as mean values (n = 9 or 10) with SD.

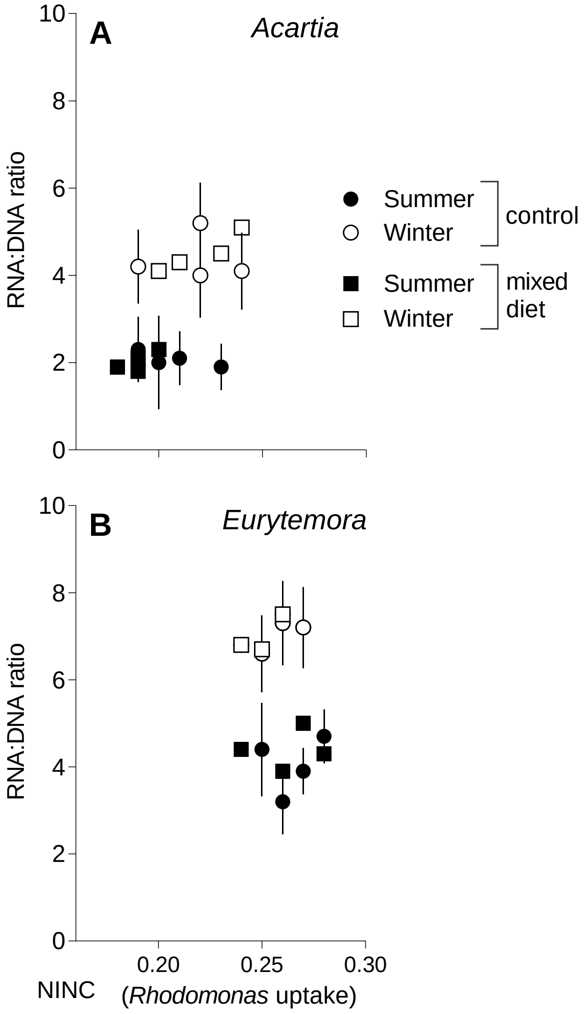

Supplement: Supplementary file 1 [file Presentation_1.pdf]
